# HOW OLDER ADULT PATIENTS PERCEIVE THE IMPORTANCE OF AMBULATION DURING HOSPITALIZATION

**DOI:** 10.1093/geroni/igae098.1791

**Published:** 2024-12-31

**Authors:** Barbara King, Linsey Steege

**Affiliations:** UW-Madison, Madison, Wisconsin, United States; University of Wisconsin-Madison, Madison, Wisconsin, United States

## Abstract

**Background:**

Older adults commonly experience loss of walking independence during a hospital stay. Loss of functional mobility is the greatest threat to independent living post discharge and quality of life. Limited ambulation during hospitalization is the most predictable and preventable cause of loss of functional mobility. There is an abundance of data on frequency of patient ambulation and the number of steps taken exists, but little information on how older adult patients perceive ambulation during hospitalization. The purpose of this study was to understand how older adult patients perceive ambulation when admitted to a hospital setting. Method: A qualitative study using inductive content analysis was conducted. Fifteen (n = 15) older adults participated in one-on-one in-depth interviews three months post discharge. Interviews were audio recorded and transcribed verbatim. Analysis consisted of open coding to identify main and supporting categories that describe the patient’s experience.

**Results:**

Value of Ambulation was identified as the main category which consisted of four supporting categories, Maintaining Health, Filling Time, Getting Ready and Being Connected. Older adults perceived walking during a hospital stay as critical for managing their acute and chronic illness, connecting with other patients and unit staff and limiting social isolation, helping fill their time to reduce boredom, and served to determine if they felt ready to go home.

**Conclusion:**

Ambulation during a hospital stay is highly valued and matters to older adults. Additional research on the patient experience with ambulation is needed to develop person-centered care to improve functional outcomes for older persons.

